# Trends, seasonal variations and forecasting of chronic respiratory disease morbidity in charcoal producing areas, northwest Ethiopia: time series analysis

**DOI:** 10.3389/fepid.2024.1498203

**Published:** 2025-01-15

**Authors:** Mulugeta Tesfa, Achenef Motbainor, Muluken Azage Yenesew

**Affiliations:** ^1^ Department of Public Health, College of Medicine and Health Sciences, Debre Markos University, Debre Markos, Ethiopia; ^2^ Department of Environmental Health, School of Public Health, College of Medicine and Health Sciences, Bahir Dar University, Bahir Dar, Ethiopia

**Keywords:** chronic respiratory disease, trend, seasonal variation, forecasting, ARIMA, Ethiopia

## Abstract

**Objective:**

This study analyzed the trend, seasonal variations and forecasting of chronic respiratory disease morbidity in charcoal producing areas, northwest Ethiopia, aiming to provide evidences in planning, designing strategies, and decision-makings for preparedness and resource allocation to prevent CRD and reduce public health burden in the future.

**Materials and methods:**

The trend, seasonal variation, and forecasting for CRD were estimated using data collected from the three zones of Amhara region annual reports of DHIS2 records. Smoothing decomposition analysis was employed to demonstrate the trend and seasonal component of CRD. The ARIMA (2, 1, 2) (0, 0, 0) model was used to forecast CRD morbidity. The model's fitness was checked based on Bayesian information criteria. The stationarity of the data was assessed with a line chart and statistically with the Ljung-Box Q-test. SPSS version 27 was utilized for statistical analysis.

**Results:**

The annual morbidity rate of CRD has shown an increasing trend in both sexes over a seven-year period among people aged 15 years and older. Seasonal variation in CRD morbidity was observed. The smoothing decomposition analysis depicted that the seasonal component was attributed to 44.47% and 19.16% of excess CRD cases in the period between September to November, and June to August, respectively. A substantial difference among the three zones of the Amhara region in CRD morbidity rate was noted, with the highest observed in the Awi zone. Forecasting with the ARIMA model revealed that CRD-related morbidity will continue to increase from 2020 to 2030.

**Conclusion:**

The study revealed that the CRD morbidity rate has shown an increasing trend from 2013 to 2019. Seasonal variation in the CRD morbidity rate was observed, with the highest peak from September to November. The morbidity attributed to CRD will continue to increase for the next ten years (2020–2030). Therefore, this study could potentially play a groundbreaking role. Further study is warranted to understand the risk factors and facility readiness through a further understanding of seasonality and future trends.

## 1 Background

Chronic respiratory disease (CRD) is a significant global health issue, causing ill health, early death, and disability in low- and middle-income tropical countries (LMICs) (1, 2). It was the third leading cause of death, next to cardiovascular disease and cancer (3). Globally, deaths of CRDs increased by 28.5% from 1990 to 2019 (4) and accounted for 7% of all forms of global deaths (5). CRD had increased nearly by 40% between 1990 and 2017 (6), and LMICs contribute to over 85% of all CRD cases (7). The burden of CRD will continue to affect hundreds of millions of people globally, with disproportionate growth in low- and middle-income regions through 2050 (8). The existing CRD burden is huge and much earlier than the predicted ranking by 2030 (9). The World Health Organization has identified the prevention and control of CRDs as an urgent development issue and essential to the achievement of the Sustainable Development Goals (10). In Ethiopia, among all modifiable risk factor-attributed deaths and DALYs of NCDs, chronic respiratory diseases contribute 41% next to cardiovascular diseases (11). Ethiopia has adopted a WHO “Best Buys” intervention to achieve the SDGs' goals and is presently executing a National Strategic Plan for the Prevention and Control of Major Non-communicable Diseases in 2020 (12).

Asthma and chronic obstructive pulmonary disease (COPD) are the most common types of CRDs and contribute significantly to mortality, morbidity, and reduced quality of life (13). Higher morbidity is common in asthma disease than in COPD, but mortality is higher in COPD than in asthma cases (14). The number of COPD cases among those aged 25 years and older will increase by 23% from 2020 to 2050 globally, and the number of cases in LMICs will be expected to be more than double that of high-income regions (8), Understanding these diseases, helps address a substantial portion of the CRD burden among adults.

CRD has been linked to several risk factors (15). Environmental exposure to tobacco smoke, indoor and outdoor air pollution, occupational dust, biomass fumes chemicals, and infections are important risk factors for COPD and asthma (16–19). Likewise, the global burden disease report revealed that decreasing exposure to environmental and occupational risks would have contributed to a 2.6% decline in DALYs from all causes, with a decline of about 6% for NCDs, including CRD (20).

Similarly, Ethiopia had an unacceptably high rate of early readmission for acute exacerbations of asthma and COPD due to these factors (21). In 2019, household air pollution was the 1st leading risk factor for all-cause death in Ethiopia (22) and it was responsible for approximately 48% of Ethiopia's LRI-related deaths across all population groups (23). Biomass is a primary energy source for cooking and heating in Ethiopia, especially in rural areas, where wood, charcoal, and agricultural waste are widely used (24). Ethiopia is one of the largest charcoal-producing countries in Africa (25). Charcoal production in Ethiopia is critical for environmental and health impacts associated with traditional practices (26). Respiratory symptoms are common among charcoal producers and users (27).

Seasonality has been demonstrated for several clinical conditions where symptoms and mortality increase in winter; for example, cardiovascular disease (28), pulmonary embolism (29), COPD exacerbations, and hospitalizations related to COPD (30, 31), are also most common in winter. Furthermore, other studies have shown that seasonal variations in asthma and COPD morbidity rates and hospitalizations were higher in the cold and pollen seasons (32–34). Although respiratory diseases have seasonal patterns, season-specific inflammatory potentials may vary from region to region (35).

A time series data is a sequence of observations taken sequentially over time (36). Time series data analysis is a valuable tool for assessing the occurrence of disease at various points in time since trends and seasonal variations impact the functioning of the health system, including CRD treatment and care (37). There are some studies in Ethiopia on the seasonal differences in the occurrence of asthma and COPD. Nevertheless, earlier research on the seasonal fluctuations in asthma or COPD morbidity rates in Ethiopia was limited to particular sites for shorter periods and was based on single-pharmacologic prescription studies (38–40).

Moreover, low self-care practice is common in most of the patients in northwest Ethiopia, which accounted for 42.3% of uncontrolled asthma (41). This is also true at the country level in Ethiopia, where 45% of asthma cases experienced uncontrolled asthma (42). Thus, it is important to understand trends and seasonal variations for asthma, COPD, and CRD in the region to target and prioritize prevention efforts. Likewise, forecasting for these diseases also helps to generate information to forewarn health institutions, healthcare providers, and decision-makers of the need for prudent decisions about resource allocation. Therefore, this study aimed to investigate the trend, seasonal variations, and forecasting of CRD, asthma, and COPD morbidity in the region using seven years of data. The results will be very helpful in planning the health care system and developing preventative methods to manage and control CRD, especially in regions where large-scale charcoal production is practiced.

## 2 Materials and methods

### 2.1 Study design and settings

A retrospective observational longitudinal study was carried out using data extracted from the three zones (Awi, East Gojjam, and West Gojjam) of the Amhara region to investigate the trend, seasonal variations, and forecasting of asthma, COPD, and CRD. The three zones are among the thirteen zones of the Amhara National Regional State, located in the southwest of the Amhara region, Ethiopia, which are situated between 9° 20′ and 14° 20′ North latitude and 36° 20′ and 40° 20′ East longitude. East Gojjam, West Gojjam, and Awi zones are located at 300, 387, and 447 km to the northwest of Addis Ababa and at a distance of 265, 176, and 118 km from Bahir Dar, the capital city of Amhara region, respectively. The data extraction was done from October to December 2022 using DHIS2 quarterly reported CRD cases to the zones during 2013–2019. Consideration was given to bronchial asthma and COPD cases in the analysis since these are the most prevalent types of CRDs (43, 44). Health-related data are compiled at the district level and reported to the Zonal, Regional, and Federal Ministry of Health through the Health through DHIS 2 data management system. We used quarterly data and the data include the number of asthma and the number of bronchitis, emphysema, and bronchiectasis to take as COPD cases in our study. Based on the 2007 national population and housing of Ethiopia, from the Central Statistics Agency, the projected population for 2022 in the study area was about 6,837,762 of which 3,410, 856 (49.9%) were male and 3,426,906 (50.1%) were female and 82% were living in rural areas (45).

The study area is well-known for its massive wood charcoal production, and it is one of the areas where large quantities of charcoal are transported to Addis Ababa annually (46). For instance, only in Mecha district (one of the districts located in the study area), around 164648.2 tons of charcoal was produced from 2014 to 2018 (47). Charcoal production is one of the major sources of households' livelihoods, as a means of generating income (48). Despite its advantage in resolving the shortage of high energy demand and serving as a source of income, the process of producing charcoal, as well as its use, exposes workers and nearby communities to various harmful pollutants, which in turn affects human health, notably the respiratory system (49).

The study area climate has four seasons: summer (a rainy season in Ethiopia from June to August), spring (September to November), Bega/winter (a dry season in Ethiopia lasting from December to February), and autumn (March to May) (50).

### 2.2 Study population, sample size, and data extraction method

Data were extracted from DHIS2 annual records of CRD (including asthma and COPD) using an extraction format prepared by the principal investigator before the actual extraction period. The data were extracted from all cases aged 15 years and older who visited public health facilities during the specified period (2013–2019). Data extraction was done at the zonal level, where the organized DHIS2 records are available. Data recorded at the health facility level is reported to the respective district health offices and the district health office reports to zonal health departments. The data for this study were collected from 44 districts in the three zones. All cases were regarded as discrete episodes, even if a patient could have revisits during this period.

### 2.3 Data quality

The data extraction tool was prepared based on the DHIS2 registry format. The data extraction guideline was prepared and pretested for its fitness to account for the required information before actual data collection. The data collectors were informed and trained about the objective of the study and how data extraction is carried out in the data collection procedure. After extraction, the data were checked for completeness and consistency by the principal investigator daily. Crosschecking was also done with the quarterly and annual reports of the districts and Amhara Regional State Health Bureau records. The data were carefully entered into Microsoft Excel for further cleaning and analysis. Initially, the dataset was screened for inconsistencies, such as duplicate records, outliers, and incorrect or misformatted entries. Errors were corrected through standardized transformations, including removing duplicate entries and correcting formats (e.g., age category formats).

### 2.4 Data processing and analysis

The annual morbidity rates for COPD, asthma, and CRD per 100,000 people were determined for each year. Multiple subgroup analyses were performed; the first group included CRD, which included both asthma and COPD; the second group included asthma, and the third group included COPD.

#### 2.4.1 Trend and seasonal variations analysis

The center of the moving average was plotted to observe seasonal, and annual fluctuations and trends of CRD morbidity (51) in the study area. A smoothing technique was used to reduce data variability that might occur in time series as a result of irregularities and seasonality to observe trends and seasonal distribution of CRD. Smoothing was performed based on the moving average window method (using a four-quarter window) (52). Excel 2019 was used to demonstrate the trend and calculate seasonal components of CRD.

#### 2.4.2 Forecasting

The intrinsic feature of time series data is that, typically, adjacent observations are dependent (36). The nature of this dependence among observations of time series is of considerable practical interest. ARIMA model (2, 1, 2) (0, 0, 0) was employed to forecast CRD for the next five to ten years using data from 2013 to 2019, where (2, 1, 2) is a stationary part of the model and (0, 0, 0) is seasonal part of the model. ARIMA model is a typical statistical technique capable of finding a fitting function in an iterative way through the Box-Jenkins procedure. The ARIMA model uses the autoregressive parameters (AR), the number of differencing passes(d), and moving average parameters (AM) to describe the series in which a pattern repeats seasonally over time (53).

Before forecasting, the stationarity of our time-series data was assessed with a line chart, P-P plot, and Ljung-Box Q-test. We consider the differencing of (*d* = 1) to transform the data to be stationary. To determine the time lags for the current observation, the autocorrelation function (ACF) and partial autocorrelation function (PACF) were used to estimate the auto-regression (AR) and moving average (MA) models. The Box and Jenkins method was used to determine the ARIMA model (36); identification of stationarity, estimation of the AR and MA models graphically using ACF and PACF correlograms, and diagnose and check the model. After the identification of the ARIMA model, it was checked using the Bayesian information criterion to evaluate the goodness-of-fit of constructed models, and a model with a BIC value of 15.67 was chosen to select the best ARIMA model to forecast CRD. SPSS version 27 was used to undertake the forecasting analysis.

## 3 Results

### 3.1 Trends of CRD, asthma and COPD

The trend of CRD has clearly shown an increase from 2013 to 2019. The average number of CRD cases during the seven years in the three zones was 30942 (95% CI: 19133, 42750). The yearly average CRD morbidity rate was 460 (95% CI: 302, 619 per 100,000 population per year, *p* trend <0.001) during the study period in the three zones. The overall CRD morbidity rate showed an increasing trend from 305 in 2013 to 731 per 100,000 population in 2019, which translates to a relative increase of 39.7% annual change.

**Figure 1:**
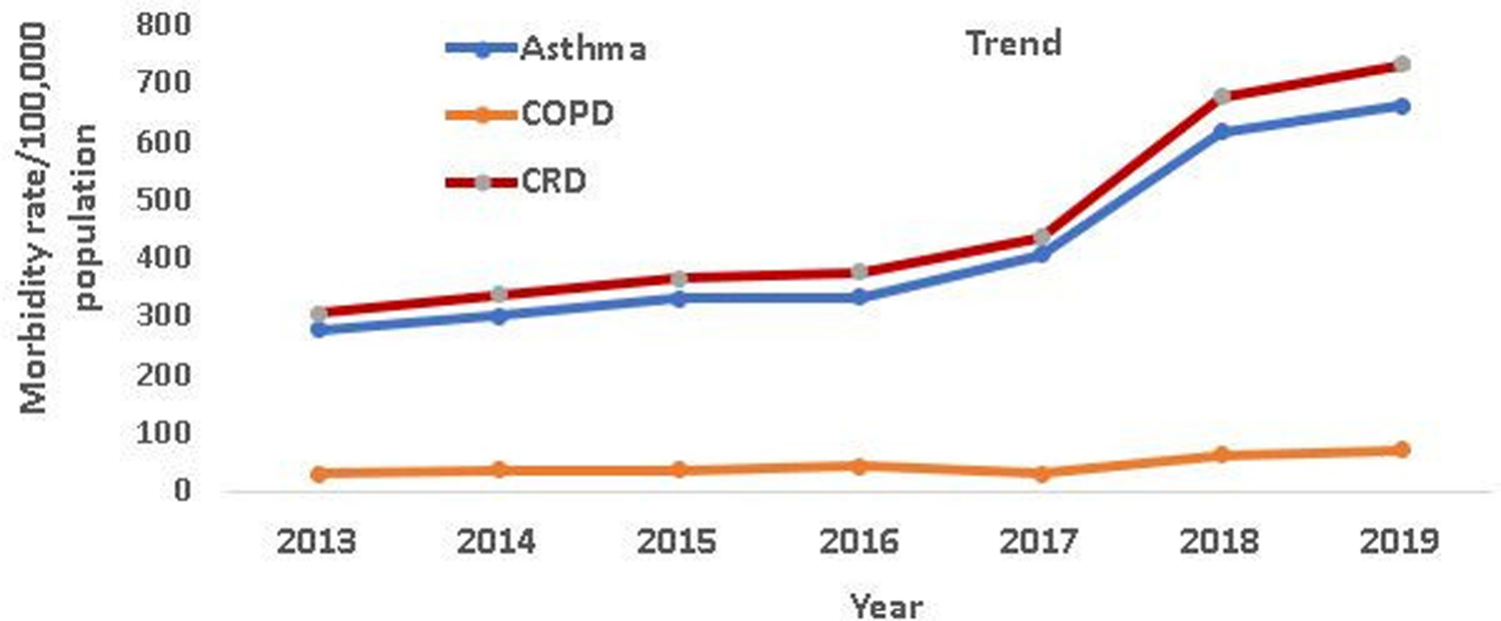
The trends of asthma, COPD and CRD morbidity rates/100,000 population from 2013 to 2019 in the three zones of Amhara Region, Ethiopia.

Among the two common CRD respiratory diseases, the number of asthma cases always occupied first place every year and accounted for 90.5% of the total CRD cases from 2013 to 2019. The overall average of asthma cases during the seven years was 28,008 (95% CI: 17,208, 38,808) in the three zones. The annual average rate of morbidity was 417 (95% CI: 277, 562 per 100,000 population per year, *p* trend <0.001). The trend of asthma morbidity rate increased from 275.6 to 661 per 100,000 population in 2013 and 2019 respectively, which translates to an increase of 39.9%. Similarly, the COPD morbidity rate also varied from 29.3 in 2013 to 69.7 in 2019 per 100,000 population, representing a 38.1% relative increase. The yearly average of COPD cases was 2,933 (95% CI: 1,841, 4,025) per 100,000 population per year, *p* trend <0.001) during the seven years in the three zones (Figure 1). The majority of asthma (58.6%) and COPD (54.9%) were reported in the 30–64-year-old group (Table 1).

**Table 1:** Summary statistics for asthma, COPD, and CRD morbidity in the three zones of northwest Amhara from 2013 to 2019.

	2013	2014	2015	2016	2017	2018	2019	Average No. of cases (95% CI)/100,000 population
No. of cases in both sexes in three zones								
Asthma cases								
Male	7,925	9,143	10,468	10,939	12,931	20,455	21,417	14,226 (95% CI: 8,614, 19,837)
Female	8,839	9,485	10,263	10,584	12,423	18,621	21,318	13,782 (95% CI: 8,575, 18,990)
Total	16,764	18,628	20,731	21,523	25,354	39,076	42,735	28,008 (95% CI: 17,208, 38,808)
COPD cases								
Male	928	1,331	1,264	1,728	1,081	1,953	2,477	1,639 (95% CI: 1,093, 2,185)
Female	853	920	1,005	1,027	828	1,954	2,030	1,294 (95% CI: 721, 1,867)
Total	1,781	2,251	2,269	2,755	1,909	3,907	4,507	2,933 (95% CI: 1,841, 4,025)
CRD cases								
Male	8,853	10,474	11,732	12,667	14,012	22,412	23,894	15,865 (95% CI: 9,798, 21,933)
Female	9,692	10,405	11,268	11,611	13,251	20,575	23,348	15,076 (95% CI: 9,322, 20,830)
Total	18,545	20,879	23,000	24,278	27,263	42,987	47,242	30,942 (95% CI:19,133, 42,750)
Age distribution of asthma and COPD cases								
	15–29 years			30–64 years			≥65 years	
Asthma								
Male	23,321			53,166			16,791	
Female	23,183			55,220			13,130	
COPD								
Male	2,260			5,811			2,691	
Female	1,810			4,825			1,982	

**Figure 2:**
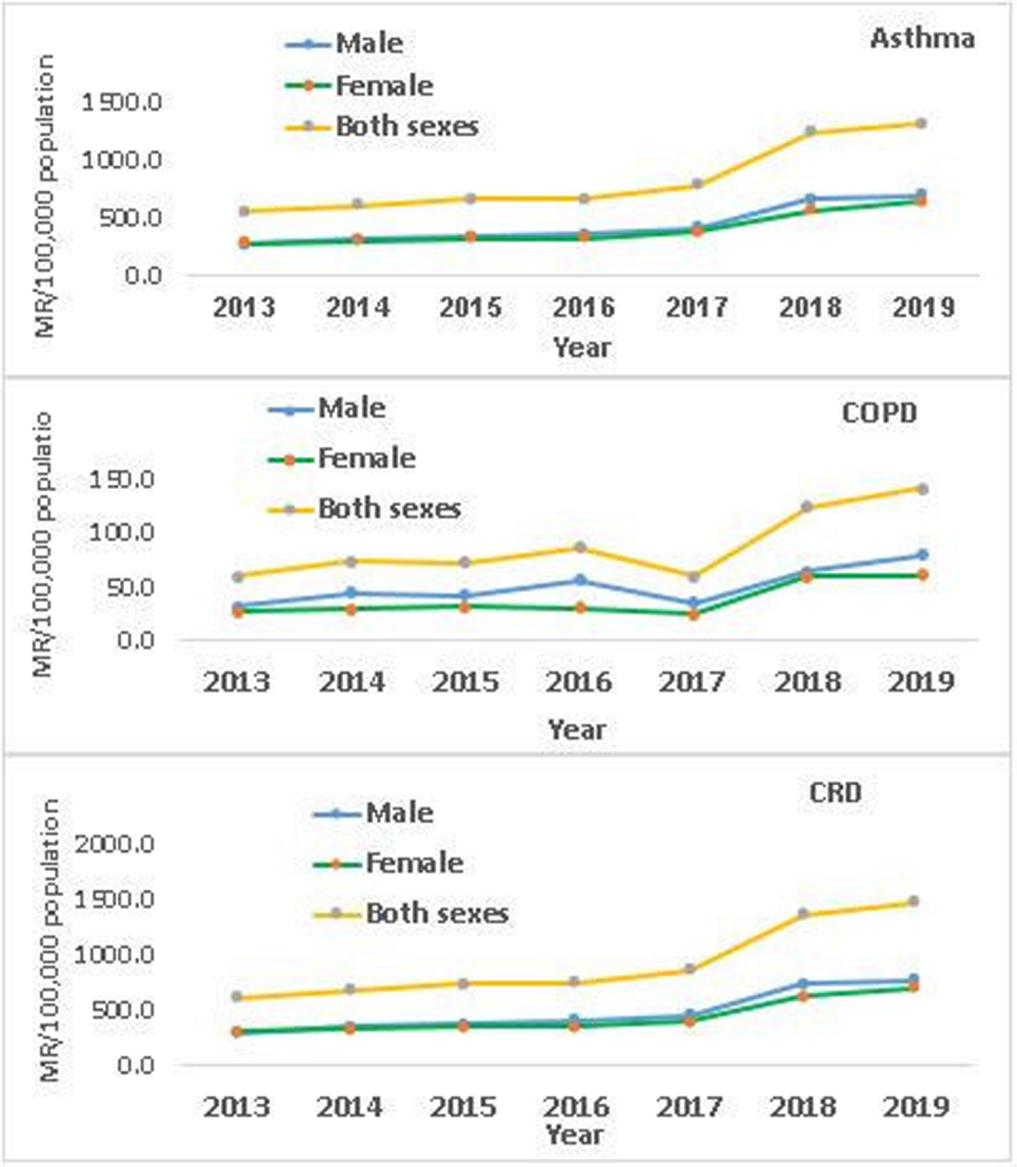
Trends of asthma, COPD, and CRD in both sexes during 2013–2019.

The morbidity rates due to asthma and COPD persistently increased in both sexes from 2013 to 2019 (Figure 2). The of male to female cases of asthma, COPD, and CRD was proportional across the seven years, even though the proportion of male cases was slightly higher. However, there is a difference in the relative percent change of asthma and COPD cases in both sexes during the study period. A relative percent change of 70% and 58% was detected in male and female asthma cases respectively. In the same pattern, a relative percent change of 67% and 38% was noted in male and female COPD cases, respectively. Overall, from 2013 to 2019, an increase in the trend of CRD in both males and females was observed across the three zones.

**Figure 3:**
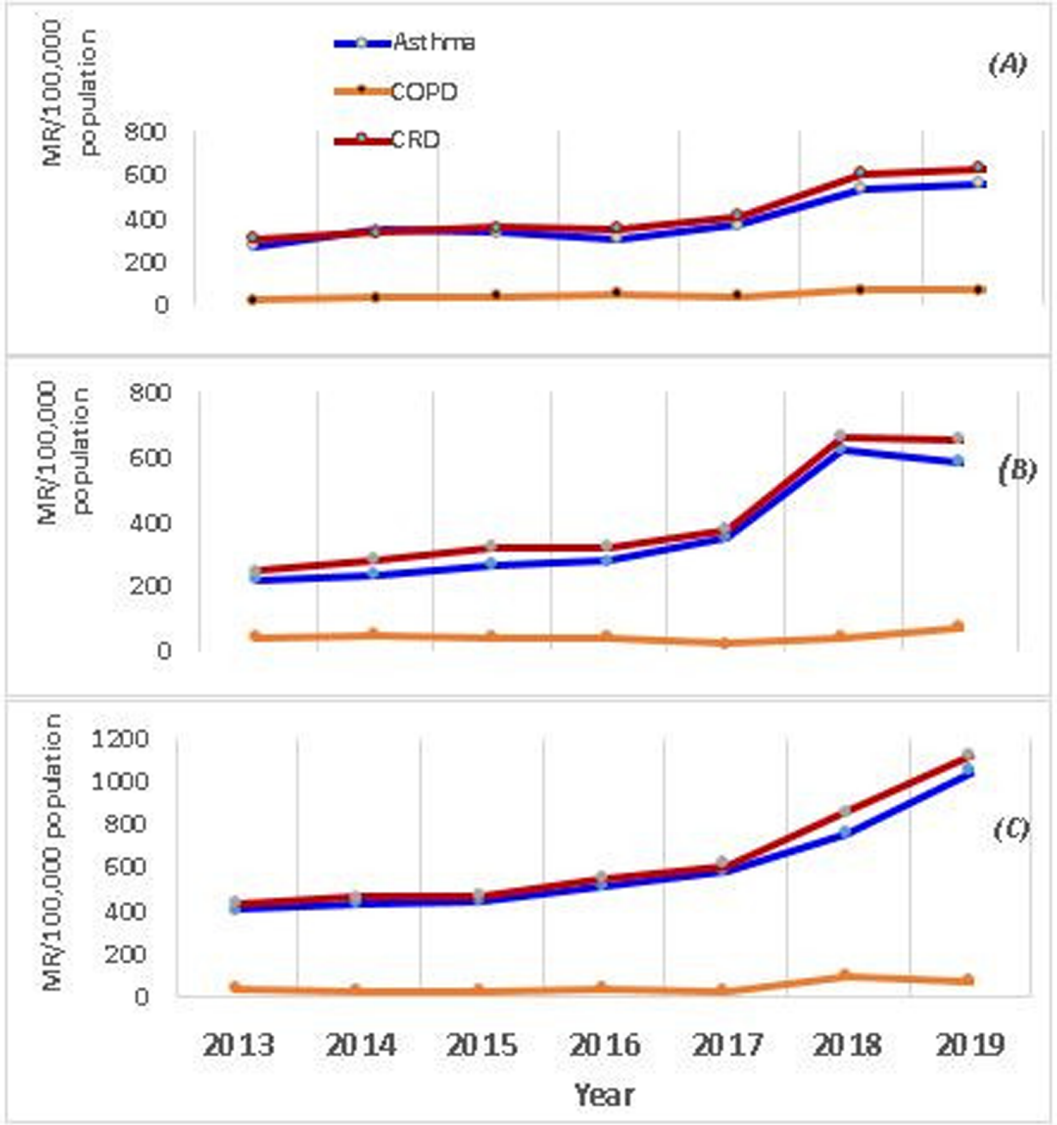
Trends of asthma, COPD and CRD during 2013–2019, northwest Ethiopia. Trends of Asthma, COPD and CRD in each zone; WGZ **(A)**, EGZ **(B)** and AWI **(C).** MR, morbidity rate.

The morbidity rates attributed to asthma, COPD, and CRD showed variations across the seven years (Figure 3). The yearly average morbidity rates of asthma, COPD, and CRD for Awi from 2013 to 2019 were 594.7 (95% CI: 382.6, 806.8), 46 (95% CI: 20, 71.8), and 640 (95% CI: 407, 873) per 100,000 population, respectively. Indeed, the relative percent change in asthma, COPD, and CRD morbidity rates from 2013 to 2019 was also 58.2%. 11.3% and 56.7% respectively.

The yearly average morbidity rates of asthma, COPD, and CRD for WGZ from 2013 to 2019 were 389 (95% CI: 285, 493), 46 (95% CI: 31, 62), and 427 (95% CI: 305, 550) per 100,000 population, respectively. Concomitantly, the relative percent change of asthma, COPD, and CRD morbidity rates from 2013 to 2019 were also 6.4%, 97%, and 7.2%, respectively.

The yearly average morbidity rates of asthma, COPD, and CRD for EGZ from 2013 to 2019 were 365 (95% CI: 209, 522), 40 (95% CI: 25, 55), and 406 (95% CI: 245, 569) per 100,000 population respectively. Similarly, the relative percent change of asthma, COPD, and CRD morbidity rates from 2013 to 2019 was 66%, 10%, and 65.8%, respectively. The peaks of yearly asthma, COPD, and CRD would reach in 2017 and desire further investigation.

**Figure 4:**
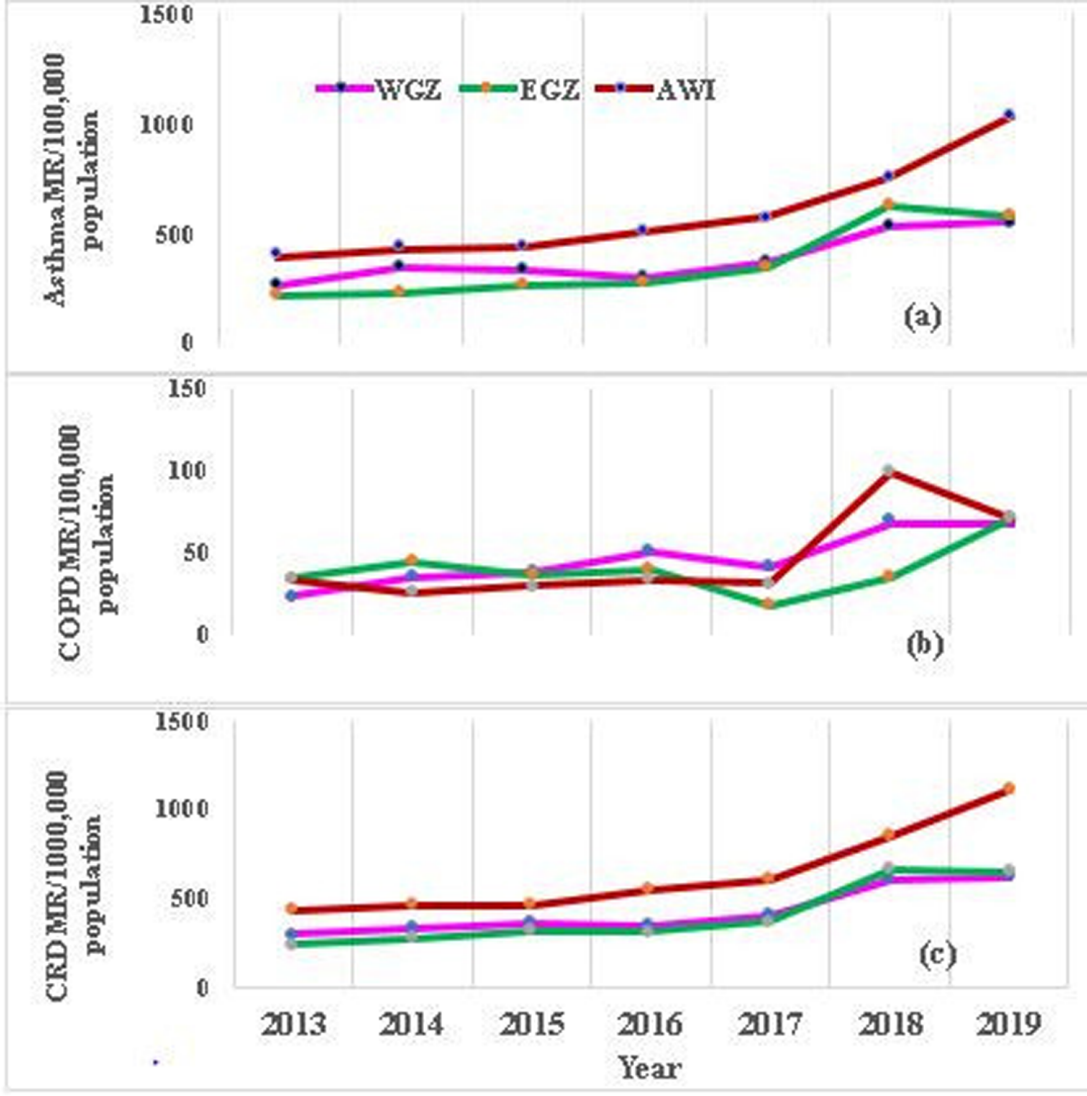
Trends of CRD, asthma and COPD morbidity rate per 100,000 population in people aged 15 years and older in the three zones of amhara region, northwest Ethiopia, 2013–2019. Morbidity rates of Asthma **(a),** COPD **(b)** and **(c)** CRD in three zones. MR, Morbidity rate; WGZ, West Gojjam zone; EGZ, East Gojjam zone.

Overall, the trends of asthma, COPD, and CRD morbidity rates during the study period are increasing year-to-year across the three zones (Figure 4). The morbidity rate from chronic respiratory disease varied greatly among the three zones. The highest cumulative average CRD morbidity rate during the seven years increment was observed in the Awi zone, at 4,508.8 per 100,000 population, followed by West Gojjam, at 3,005.8 per 100,000 population, and East Gojjam, at 2,905 per 100,000 population. However, there was some overlap between the East and West Gojjam zones in CRD morbidity rates in the year 2018.

### 3.2 Seasonal variations of CRD

Asthma, COPD, and CRD have clearly shown seasonal patterns in the three zones during the study period. The proportions of summer, spring, winter, and autumn CRD cases were 59,189 (29%), 71,944 (35%), 39,297 (19%), and 33,764 (17%) respectively. The months from June to August and September to November had a higher morbidity rate for asthma compared to the remaining months of the year. A peak was observed for COPD from September to November and December to February compared to the rest of the months of the year. This seasonal pattern was detected throughout the seven years (Table 2).

**Table 2:** Seasonal variation of CRD morbidity in the three zones of northwest Amhara, Ethiopia from 2013 to 2019.

Year	June to August	September to November	December to February	March to April
2013	6,199	7,313	2,474	2,559
2014	6,235	7,203	3,681	3,760
2015	6,561	7,514	5,044	3,881
2016	7,971	8,213	4,350	3,744
2017	8,458	10,185	4,389	4,231
2018	12,208	14,693	8,719	7,367
2019	11,557	16,823	10,640	8,222
Total	59,189 (29%)	71,944 (35%)	39,297 (19%)	33,764 (17%)

**Figure 5:**
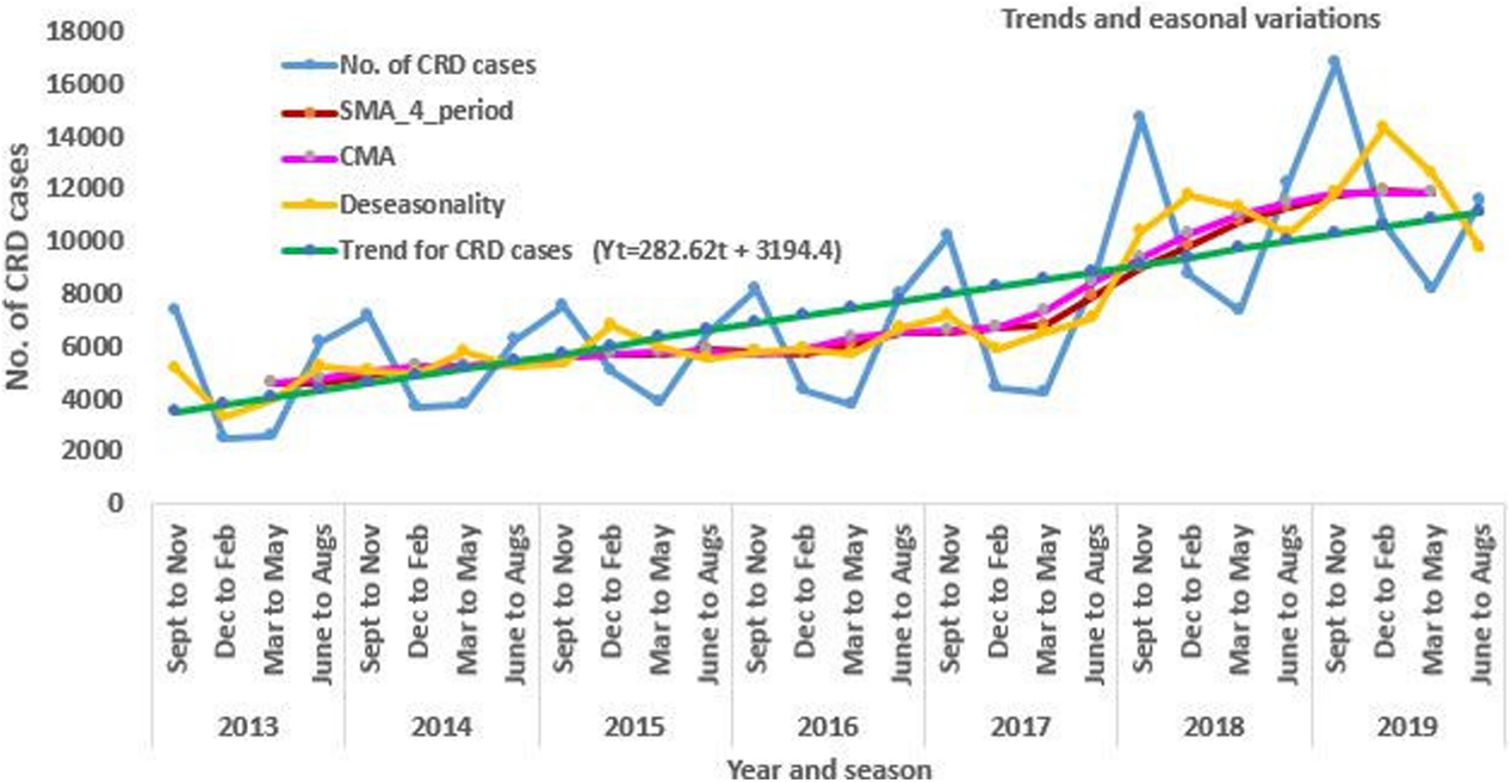
The trend and seasonal variations of CRD morbidity in the three zones of Amhara region, Northwest Ethiopia from 2013 to 2019.

Calculating a moving average at specific intervals smooths out the data, by reducing the impact of random fluctuations. The result of smoothing using the moving average indicated that the seasonal component attributed to 41.47% (with a seasonal index value of 1.4147) and 19.16% (with a seasonal index value of 1.1916) of excess CRD cases in the period between September to November and June to August, respectively, above the cycle mean. However, in the period between December to February and March to May, on average 28.8% and 34.76% below the base of the cycle mean respectively. The observed ascending trend is statistically significant (*p* < 0.001) (Figure 5).

### 3.3 Forecasting of CRD

**Figure 6:**
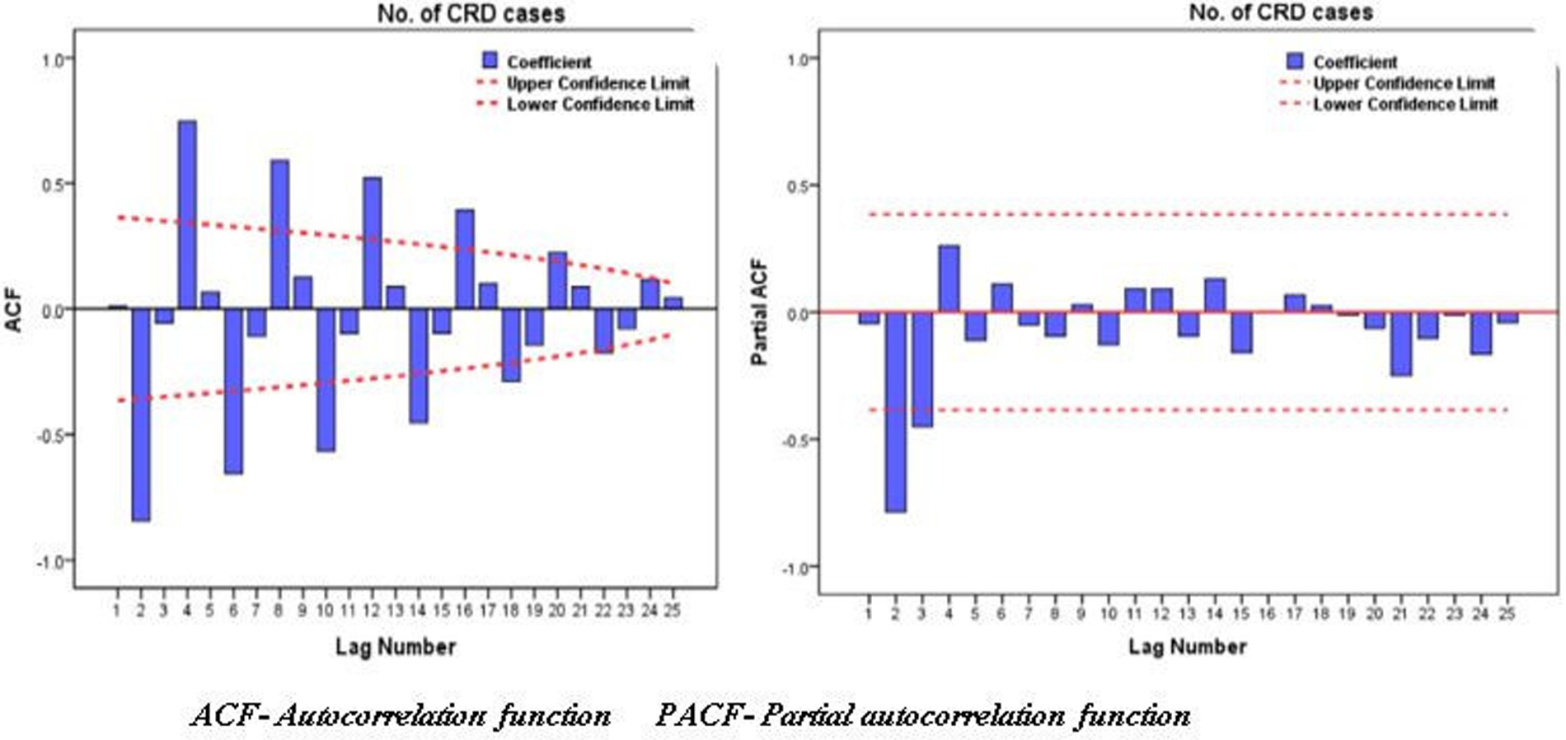
ACF and PACF plot of CRD cases during 2013–2019 with transformation of at differencing of one (*d* = 1).

**Figure 7:**
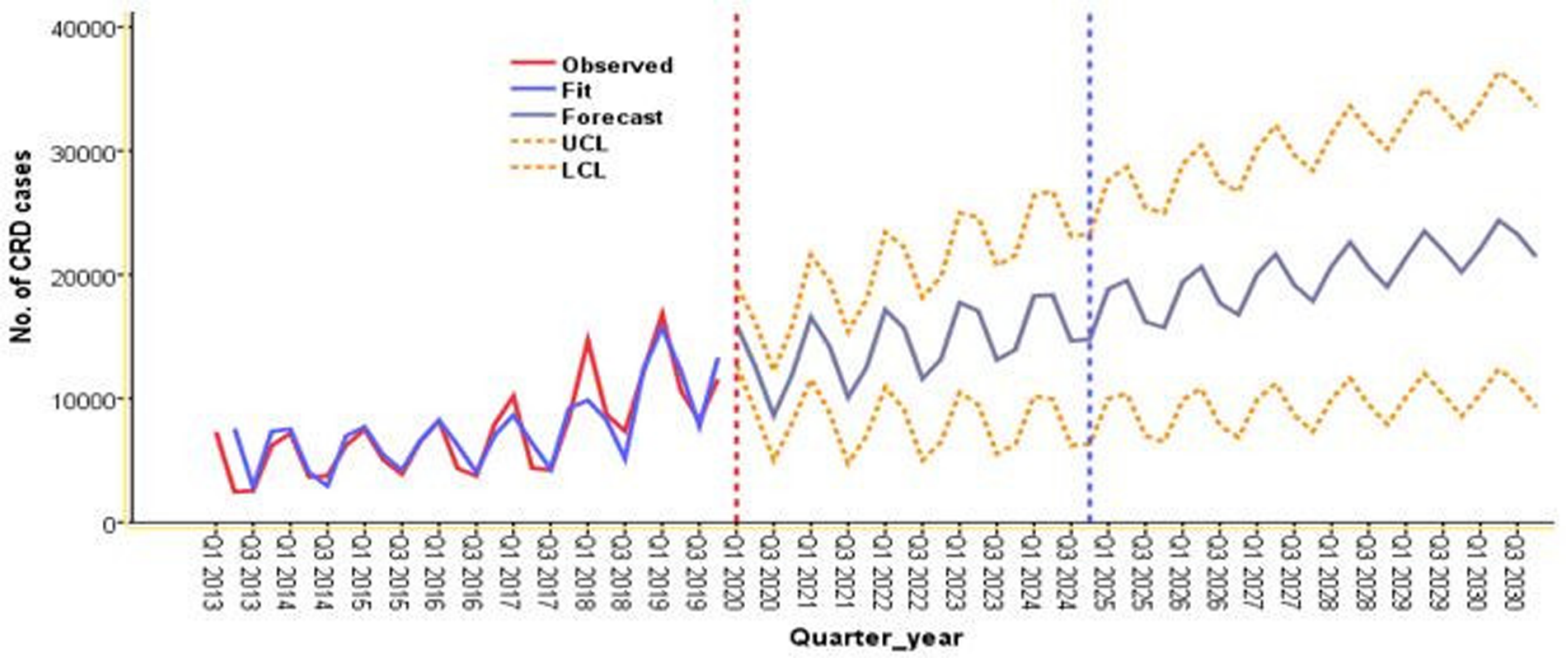
Forecasted CRD for five to ten years (2020-2024-2030) using data from 2013 to 2019 in the three zones of Amhara region, northwest Ethiopia.

The trend of CRD was forecasted for the next five to ten years (2020-2024-2030) based on the 2013–2019 data. Since the data clearly showed trend and seasonality, we used differencing to maintain the stationarity. The ARIMA model (2, 1, 2) (0, 0, 0) was utilized to forecast the CRD pattern based on the values estimated from the ACF and PACF plots (Figure 6). The result from forecasting revealed that in the next five to ten years, the trend of CRD will increase by 39.2% and 38% from 2020 to 2024 and 2024–2030, respectively (Figure 7). Similarly, the trend of asthma and COPD will increase by 38.2% and 24% from 2019 to 2024 and by 41.4% and 39.9% from 2024 to 2030, respectively.

## 4 Discussion

This study aimed to investigate the trends, seasonal variations, and forecasting of asthma, COPD, and CRD in the three zones of the Amhara region.

The study showed a concomitant annual increase in the morbidity rate of asthma and COPD, which subsequently resulted in an increase in CRD across seven years, which is consistent with reports from previous studies (54, 55). The study also revealed that morbidity due to asthma was found to be the highest proportion (95.5%). Similar results have been noted in prior studies conducted elsewhere (43, 44). This may suggest that potentially asthmatic cases were seeking care to reduce their wheezing symptoms. However, a large proportion of deaths attributed to CRD accounted for COPD (4, 56). COPD causes morbidity and mortality in adults in SSA (44). Evidence from earlier research has indicated that exposure to pollutants attributed to particulate matter arising from various sources is the major contributor to deaths from COPD in areas with a low socio-economic index (5, 38). In contrast, asthma has been reported as a common cause of death in Nigeria (57).

The current study also identified that the morbidity rates due to asthma, COPD, and combined CRD persistently increased in both sexes across the seven years. In addition, the morbidity rate is almost similar in both sexes, which is similar to the result observed in Tanzania (58). However, in contrast to the present study, the study in India revealed that females outweighed males (59). The difference might be because the present study used secondary data from DHIS 2 records of three zones, including zonal and district towns, and may have the potential to use clean fuel, in comparison to the previous study conducted in a rural village where biomass fuels meet their daily heating and cooking.

Interestingly, the study revealed that the morbidity rate due to asthma, COPD, and combined CRD varied among the three zones. The yearly average morbidity rate of asthma and CRD was highest in the Awi zone compared to WGZ and EGZ. Nevertheless, the relative percent change in asthma morbidity rate was the highest in EGZ. Whereas, the highest relative percent change in morbidity rate attributed to COPD was identified in WGZ. This suggests that there is a significantly increased number of asthma and COPD cases observed in EGZ and WGZ respectively in 2019 compared to 2013. The estimated peak-to-trough difference was higher for asthma than for COPD. Studies have also demonstrated that geographic variation in CRD morbidity rates may be linked to an increase in environmental risk and low socioeconomic factors (60, 61, 62, 63). In that context, it will be necessary to understand the factors influencing the higher morbidity rate in Awi compared to the others in future studies.

The study found that trends in asthma, COPD, and CRD increased steadily across the three zones during the past seven years in adults aged 15 years and older. This is in agreement with trend studies conducted elsewhere (43, 64, 65). This may reflect increased exposure to indoor and outdoor pollution and occupational exposure to risk factors such as charcoal production and agricultural activities, climatic variability, and exposure to motor vehicle gases and other chemicals (8, 66). Despite findings from some studies indicating a downward trend in COPD mortality (67), the trend of morbidity rates increased (68). Earlier research revealed that CRD is mainly attributed to occupational risk factors (69), and 2% of the world's disease burden is attributed to biomass smoke exposure (70).

In the current study, trends were identified in asthma and COPD, with a relative increase of 39.8% and 37.9%, respectively. This is consistent with other prior research conducted elsewhere (71) and with the global burden of disease report (4, 6). It has been reported that the COPD burden in LMICs vs. HICs is expected to grow, with a projected growth of 32.7% in LMICs and 3.8% in HICs from 2020 to 2050 (8), In contrast, another study indicated that the trend of mortality due to COPD decreased in both sexes throughout 1980–2017 (72). This may be due to the use of bronchodilators, as well as enhanced knowledge of the disease, its control, and the management of its comorbidities (73).

When looking at trends by sex, both males and females CRD, COPD, and asthma trends increased persistently, despite, the relative percent change of male asthma (70%) being higher than female asthma (58%) during 2013–2019. This implied that the number of male asthma cases increased largely as compared to female cases. Similarly, the CRD trend increased in both males and females from 2013 to 2019. Correspondingly, a relative percent change of 67% and 38% was detected in male and female COPD cases, respectively. This is in agreement with a study conducted in Brazil (74). Congruently, a global study showed the trend of male cases of COPD will continue to be higher than female through 2050 (8). Differences in percent change of asthma, COPD, and CRD trends between males and females might also be partially attributed to the outdoor exposure to risk factors increased rapidly among males compared to females. In contrast, Pelkonen and colleagues in their long-time repeated cross-sectional study in Finland reported that the trend of asthma prevalence was higher in females than males (75), and COPD mortality among females increased across the study period (76). Thus, further study is advised to examine the factors linked to the disparities in illness burden between males and females.

Furthermore, our study has demonstrated that a change point in the morbidity trend of asthma and CRD increased in a steep pattern during 2017, and this permits further investigation. However, among all the years, the lowest percent change was detected from 2018 to 2019. This might be due to the time when the early phase of COVID-19 and people may be less likely to visit hospitals owing to fear of the virus, which could contribute to the drop in CRD during this time.

Despite this, the occurrence of CRD morbidity rate was rising in the three zones of the Amhara region across seven years, and the highest was observed in the Awi zone since 2017 compared to the other two zones. The reason for this is unknown, and the reasons for notably different morbidity rate variations in the three zones are worth further investigation. Further analysis of climate, including humidity and precipitation data in the different zones in the region, will help better characterize the region, and potentially shed light on the effects of weather on exacerbation rates.

Results of the present study have clearly shown marked seasonal variations in asthma, COPD, and CRD morbidity during the seven years. Of all asthma and CRD morbidity, the highest proportion of cases, 36% and 35% respectively were noted in “Tsedey” (Spring; September to November—cold season) followed by “Kirmet” (summer; June to August- heavy rainy fall) with proportion of 30% and 29% of asthma and CRD cases respectively. This is in agreement with previous studies, in which the cold season had the highest asthma exacerbations and hospitalizations (31). The reasons for the increase in CRDs with these seasons are not entirely explained in this study. However, evidence from previous studies revealed that the underlying mechanisms for morbidity during these seasons are thought to include some combination of infectious, allergic, environmental, and climatic stimuli (30, 63). The highest proportion of COPD cases (31%) was marked in winter (December to February- dry season) followed by spring with a proportion of 30%. This is in agreement with a previous study in which summer (dry season) had the highest proportion of severe exacerbations of COPD (34).

Moreover, in the current study, we noticed a 1.8-fold increase in asthma, a 2-fold increase in CRD morbidity in Spring and summer, and a 1.7-fold to 2-fold increase in COPD morbidity in winter compared to Spring, which is consistent with another study conducted elsewhere (31). Furthermore, the results of the present study illustrate that major peaks of CRD in each year occurred frequently from September to November and June to August. This seasonal component had a notable effect with seasonality indices 41.47% and 19.16% of excess CRD cases in the period between September to November and June to August, respectively, above the base of moving average, and stayed low in December to February and March to May with seasonality indices 28.8% and 34.76% below the moving average respectively. The above difference could be attributed to the differences in climatic characteristics between seasons.

Seasonal differences occurred during all seven years, indicating that the results were not the consequence of a single summer and spring. The spring and summer seasons in the present study area are characterized by the presence of cold temperatures, heavy rainfall, and high plant pollen, whereas the winter season is characterized by dry and hot temperatures. The observed differences between the seasons may highlight the importance of cold weather conditions and allergens attributed to plant pollen. Generating evidence on the seasonality of CRD contributed a significant role in early prevention and control of the disease since it would strengthen the low self-care practice in most of the patients in northwest Ethiopia (41).

The present study demonstrated that in the next five to ten years, the trend of CRD will increase by 39.2% and 38% from 2020 to 2024 and 2024–2030 respectively. This confirms a study that reported CRD tended to increase globally (13). Congruently, a previous study reported that the COPD burden is expected to grow, with a projected growth of 32.7% in LMICs from 2020 to 2050 (8).

This study has some limitations worth acknowledging. Even though the trend models show a good agreement with the reported data, we did not make any claims to take account of CRD cases who couldn't visit health facilities (in the community), this might under-estimate CRD morbidity rate in the three zones since this is a retrospective study and used aggregated data in these charcoal producing areas. In addition, to the retrospective nature of the study, it lacks information on potential environmental and lifestyle factors. Despite these limitations, we were able to draw important conclusions on trends, seasonal variations, and forecasting of asthma, COPD, and CRD morbidities over the period. We also forecasted the pattern for the next ten years in the charcoal-producing areas of the Amhara region, in northwest Ethiopia. This information would stimulate support for action to customize efforts to reduce the burden of chronic respiratory diseases.

## 5 Conclusion

The study revealed an increase in the trend of asthma, COPD, and CRD morbidity rates across seven years. The prediction also suggested that CRD will continue to increase in the next ten years in the three zones of the Amhara region, and this may result in a significant societal and healthcare burden. Furthermore, the result of smoothing using the moving average also demonstrated that, the seasonal component attributed to an excess of CRD morbidity above the base of moving average in the period between September to November and June to August. Significantly, more cases of asthma were observed during the spring (September to November) and summer (June to August). Additionally, more cases of COPD were noted in the winter. Asthma accounts for a substantial proportion of CRD morbidity compared to COPD in the study area. These findings confirm the importance of understanding periods of high risk for asthma and COPD. This also helps with readiness and planning. Interventions targeted at preventing CRD morbidity (asthma and COPD) should be initiated before the rainy and cold seasons in the study area. The community, patients, and clinicians should be fully aware of the increased risks of COPD and asthma morbidity in spring (cold season) and summer (rainy season) to enable appropriate prevention strategies to be implemented. Therefore, illness prevention awareness should raise the attention of both individuals and the relevant government departments. Future respiratory disease research studies are required to obtain specific and detailed data at the population level to discover the possible risk factors for CRDs in these charcoal-producing areas.

## Data Availability

The datasets analyzed in this study are available upon reasonable request from the corresponding author.
